# The effect of temperature on organic carbon degradation in marine sediments

**DOI:** 10.1038/srep17861

**Published:** 2015-12-07

**Authors:** Alberto Malinverno, Ernesto A. Martinez

**Affiliations:** 1 Lamont-Doherty Earth Observatory, Columbia University, Palisades, NY, USA; 2 Department of Earth and Planetary Sciences, University of California, Berkeley, CA, USA

## Abstract

The degradation of sedimentary particulate organic carbon (POC) is a key carbon cycle process that fuels the deep subseafloor biosphere. The reactivity of POC is expected to decrease with increasing sediment age, severely restricting the energy available to microorganisms. Conversely, increasing temperatures during burial have been proposed to stimulate POC degradation, possibly supplying significant energy to the deep biosphere. To test the importance of temperature, we assembled POC measurements in two global sets of drill sites where sediments underwent either relatively low or high temperatures during burial, which should have resulted in different rates of POC degradation. For ages 5–10 Ma, the decrease of the average POC content with burial is clearly more pronounced in the sites with high temperature histories. Our results support the hypothesis that temperature is one of the fundamental controls on the rate of POC degradation within deeply buried marine sediments.

The burial of particulate organic carbon (POC) in marine sediments is an important carbon cycle process that transfers reduced carbon from rapidly exchanging surface reservoirs (ocean, atmosphere, and biosphere) to the solid Earth^1^. A substantial fraction of this carbon is decomposed during sediment burial, resulting in a characteristic sequence of microbially-mediated diagenetic reactions such as sulfate reduction and methanogenesis^2^,^3^. POC degradation is the fundamental source of energy for the subseafloor biosphere^4^,^5^,^6^ and plays an important role in the global carbon cycle, as its extent controls the balance between the amount of organic carbon that is ultimately buried over geological time scales until the sediment is subducted or eroded versus the amount of dissolved inorganic carbon that is produced by POC degradation and that fluxes back into the ocean^7^,^8^. Also, POC degradation supplies the microbial methane that forms most of the vast gas hydrate reservoir in the world continental margins^9^. Gas hydrates are a unique component of the carbon cycle, as they are a metastable carbon store that can release greenhouse gases to the ocean and atmosphere, causing large climatic perturbations^10^. Warming events have been proposed to result in gas hydrate dissociation and large greenhouse gas emissions in the geologic past^11^ and they could intensify the present warming trend in the future^12^,^13^. To address the fundamental questions of how microbial cells in the deep biosphere can survive with extremely limited energy sources^5^, how much carbon is buried for geological time scales, and how to predict the occurrence of gas hydrates^14^,^15^, it is necessary to quantify under what conditions and on what timescales different POC fractions are degraded during burial. Yet, our understanding of POC degradation is still incomplete.

As POC is a mixture of organic matter types with different reactivity, during burial the most labile components are consumed near the seafloor and the overall reaction rates are expected to decrease with increasing sediment age^16^,^17^. In this view, most microbial activity is concentrated near the seafloor. Yet, deep microbial activity is demonstrated by maxima in dissolved metabolism products (inorganic carbon, ammonia, methane) hundreds of meters below the seafloor^18^ and by large numbers of active microbes in sediments older than 10 Ma^19^,^20^. Moreover, if most microbial methane is generated at shallow burial depths, the resulting predicted gas hydrate amounts are substantially lower than those observed, and it has been suggested that methane must have migrated upward from deep sources^21^. However, methane in these hydrate deposits is typically microbial, and it is unclear how it could be generated at substantial depths if POC reactivity steadily decreases with increasing age.

POC degradation at depth could be promoted by the temperature rise during burial due to the local geothermal gradient^19^. Incubation experiments show increased microbial activity at higher temperatures, and deep generation of acetate in pore waters has also been interpreted as due to the temperature increase^22^,^23^. Reaction-transport modeling shows that observed methane hydrate occurrences can be explained by deep POC degradation driven by increasing temperature^24^ and suggests that higher ocean temperatures in the Paleogene enhanced microbial methane production, building up a sizable global gas hydrate reservoir^25^. On the other hand, others found that including the temperature effect resulted in modeled concentrations of dissolved metabolites (inorganic carbon and ammonium) higher than those observed^15^. These authors concluded that temperature does not significantly affect POC degradation in deeply buried sediments, perhaps because the succession of microorganisms in the sediment column have adapted to the local temperature. While near-seafloor sulfate reduction rates increase at a given location with seasonal temperature increases^26^, the observation of similar sulfate reduction rates in cold and warm environments suggests that microbes can adapt to permanently low temperature^27^. The effect of seasonal temperature variations cannot be immediately extrapolated to long-term temperature increases such as those induced by burial^6^, and the role of temperature in POC degradation within deeply buried sediments is still unsettled.

Here we take a different approach from previous studies, which focused on experiments at laboratory time scales and on observations and modeling at a few sites, by looking at a large global data set of POC, temperature, and sediment age collected in deep sediments by scientific ocean drilling. The first step in POC degradation is a microbially-mediated depolymerization that produces dissolved organic compounds^28^. This process is generally considered rate-limiting for POC degradation, because most of the resulting dissolved compounds are rapidly metabolized^29^,^30^. Hence, for steady-state deposition, progressive degradation leads to a decrease in POC content with increasing sediment age, and this decrease depends on the reaction rates of degradation^16^,^17^. To test for the effect of temperature on long-term POC degradation, we compare POC content measured in many globally distributed drill sites where deeply buried sediments experienced different temperature histories. If temperature is important, the POC decrease with age should be more pronounced in locations where temperatures were higher during burial and degradation rates were faster.

## Results

### Globally averaged POC versus age

Our study is based on the large global data set collected in more than four decades of deep scientific ocean drilling, which includes sedimentary POC content (dry weight fraction), *in situ* temperature measurements, and age estimates. The age data were used to construct age-depth models that give sediment age and sedimentation rate at each POC data point (see Methods).

Ocean environments of POC deposition and degradation are between two extremes^3^. In areas of low biological productivity such as mid-ocean gyres, sedimentary POC is extremely low and is completely degraded by oxygen consumption. At the other extreme, in high-productivity areas (e.g., continental margins), there is enough POC in the sediment to exhaust sulfate within the first few tens of meters below the seafloor and to generate methane at depth. These differences are reflected in microbial cell counts within deep sediments, which are several orders of magnitude higher in high-productivity areas^31^.

**Figure 1:**
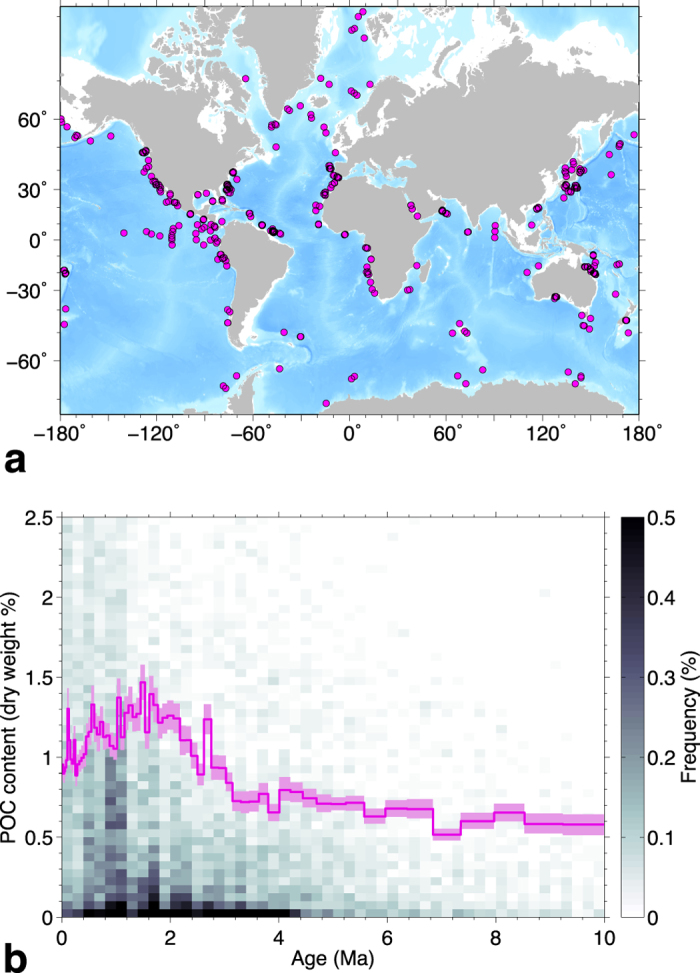
Drill sites in regions of high POC deposition. Location of 419 sites (**a**) and POC content as a function of sediment age (**b**). The background image in (**b**) is a two-dimensional histogram of 16923 data points showing the broad range of measured POC values at various ages. The continuous curve in (**b**) is the average POC calculated in age intervals that contain the same number of data points (~250). This procedure ensures that the POC averages are not affected by a variable number of data points in each age interval. The colored band around the average in (**b**) is a measure of uncertainty (68% central interval in bootstrap resampling; see Methods). The map in (**a**) was produced with the Generic Mapping Tools (GMT) package^59^.

We concentrate here on ocean regions of high POC deposition, which include continental margins and areas of upwelling, by selecting drill sites that were either within 700 km of the coastline of a land region (continents and islands with an area >2000 km^2^) or that had a POC content at the seafloor of at least 1% (interpolated from the global compilation of ref. 32). We excluded from our compilation sites in the Mediterranean Sea, which contain a unique series of Plio-Pleistocene sapropels^33^,^34^. We found 419 drill sites that are in regions of high POC deposition and have a reliable age model (Fig. 1a). Most of these sites are located on continental slopes and rises. As drilling in shallow water from a floating platform is problematic, scientific ocean drilling undersampled continental shelves, which have been the dominant locations for marine POC deposition in the Holocene^35^,^36^. Nonetheless, deeper-water locations on continental margins have lower sedimentation rates than the shelf and offer the best chance to sample by drilling the archive of older sediments that is crucial for our study.

If the POC mass fraction deposited at the seafloor is constant with time, its progressive decrease with sediment age depends only on the degradation reaction rate. The POC content of marine sediments, however, is influenced by a number of factors besides degradation^6^. The POC mass fraction generally increases with increasing sedimentation rates^37^,^38^ due to a reduced exposure time of sediments to oxic conditions^4^ and decreases only at very high sedimentation rates because of clastic dilution^36^,^39^. POC content varies during glacial/interglacial cycles, with higher POC in sediments deposited during glacial intervals, possibly because of the destruction of nearshore depositional environments by lowered sea level^37^ or of changes in the intensity and location of upwelling^40^. Sediment lithology also impacts POC content, as fine-grained material with greater surface area protects POC from rapid degradation^36^,^41^. Finally, POC measurements are of variable quality and are contaminated by errors (see Methods).

To examine whether the POC content of sediment decreases systematically with increasing age because of degradation, we average POC versus age on a global scale to attenuate the effect of measurement errors and of other variables that affect POC content (sedimentation rate, lithology, etc.). A previous global survey of ocean drilling data acquired before mid-1981 showed a trend of decreasing POC content with increasing burial depth^42^, suggesting that this is a productive strategy. The variation of the globally averaged POC content with age will mostly depend on two factors: (1) global temporal changes in POC deposition and (2) POC degradation.

The globally averaged POC versus age (Fig. 1b) increases from a seafloor value around 1% to an average of about 1.3% in the 1–2 Ma interval, followed by a steady decrease reaching a nearly constant value around 0.6% at ages >5 Ma. The POC measurements span a broad range, from a majority of small values (few tens of a percent) to few occasional high values (>1%; see the background image in Fig. 1b). These large fluctuations are the main source of uncertainty in the globally averaged POC. For example, the overall POC variability at any age is much larger than the uncertainties in the POC measurements (see Methods). We evaluated the uncertainty of the average POC computed in an age interval with a bootstrap resampling method^43^ (see Methods). The uncertainties of the computed POC averages (plotted in Fig. 1b as the central 68% interval, or ±1*σ* for a normal distribution) are generally below 0.1% despite the wide span of the measured POC values.

The increase in the average POC from the present to ~1.5 Ma points to a time-dependent deposition of the POC weight fraction. We speculate that this difference may be due to an increase in the overall sediment accumulation rates in the last few Ma^44^, which has been related to intensified erosion during glacial times^45^. If the accumulation rate of clastic components increased more than that of POC, the POC fraction of dry sediment would correspondingly decrease. Testing this suggestion requires calculating accumulation rates from the sediment porosity at each site, and is beyond our scope.

The long-term POC decrease beyond ~2 Ma may be due to long-term degradation. For example, similar POC decreases over ~10 Ma in Philippine Sea drill sites were attributed to deep degradation^46^. Alternatively, the decreasing average POC at ages >2 Ma could reflect a global temporal variation in the POC weight fraction deposited at the seafloor.

### Average POC versus age in sites with different temperature histories

To test whether temperature significantly affects POC degradation, we compare the POC content versus age in two sets of sites that differ in having experienced relatively low and high temperatures during burial. As these two sets of sites have a worldwide coverage that is comparable and fairly uniform, they should record a similar global temporal variation in the POC deposited at the seafloor. Whatever is the global history of POC deposition, if temperature is important sites in the high-temperature set should have higher reaction rates and hence display a more marked POC decrease with increasing age.

**Figure 2:**
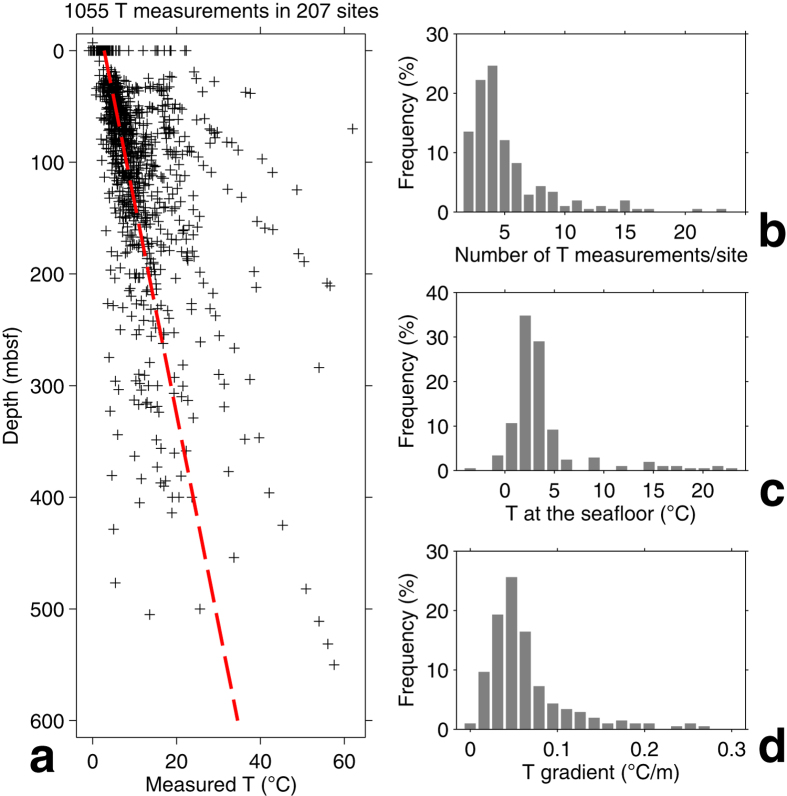
Summary of temperature measurements. *In situ* sediment temperature measurements taken in 207 drill sites (**a**) and histograms of the number of temperature measurements per site (**b**), of the temperature at the seafloor (**c**), and of the geothermal gradient at each site (**d**). Seafloor temperature and geothermal gradient were estimated with a least squares linear fit to the data (see Methods). The dashed line in (**a**) is the linear geothermal gradient for the median seafloor temperature (2.67 °C) and the median gradient (0.053 °C/m) in all the sites. Seven temperature measurements taken at depths >600 mbsf are not shown in (**a**), and four temperature gradients >0.3 °C/m are not shown in (**d**).

The formation temperature data we use are summarized in Fig. 2. Out of the 419 drill sites with an age model, 207 have reliable temperature measurements that describe a linear geothermal gradient (see Methods). It would seem at first that sites with different temperature histories during burial could be distinguished on the basis of the local geothermal gradient only, but sedimentation rate is also important. For example, consider two sites where sedimentation rates and geothermal gradients remained constant in time. If the geothermal gradients were the same but sedimentation rates were different, coeval sediments would be buried deeper and experience higher temperatures in the site that had the higher sedimentation rate. In other words, higher sedimentation rates have the same effect as a higher geothermal gradient.

**Figure 3:**
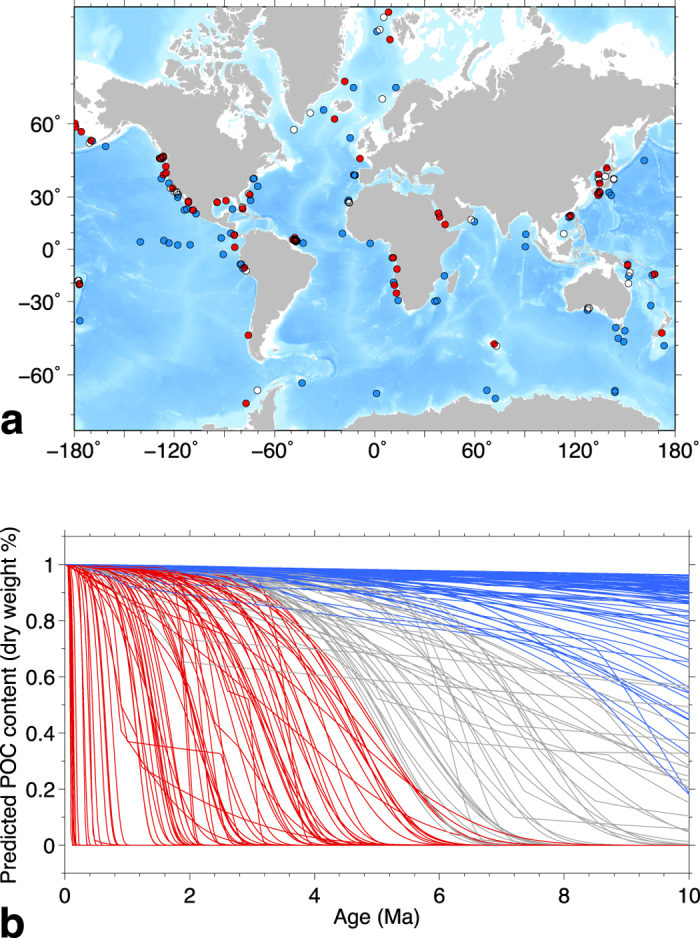
Sites with a defined local temperature gradient. Location of 207 drill sites (**a**) and predicted POC versus age *G*(*t*) from the burial temperature history (**b**), starting from 1 at the seafloor and reaching zero once all the labile POC has been degraded (see Methods). The 82 sites that experienced the lowest temperature histories are shown in blue and the 83 sites with the highest temperature histories are in red. The map in (**a**) was produced with the Generic Mapping Tools (GMT) package^59^.

To account for the effect of both geothermal gradient and sedimentation rate, we calculated the variation in the labile POC content predicted at each site by a model with temperature-dependent degradation rates. The model assumed an Arrhenius rate law, steady-state POC deposition at the seafloor, and a constant local temperature gradient in the last 10 Ma (Fig. 3b; see Methods). We stress that these assumptions are at best rough approximations. POC deposition and the geothermal gradient may have varied with time at a given location, and reaction rates may depend on factors other than temperature such as POC composition (e.g., terrestrial vs. marine). While we do not propose this simple model as a general representation of POC degradation, we use it as an effective way to quantify the hypothesized effect of temperature on POC degradation and to rank sites on the basis of the temperature histories experienced by sediments.

The predicted POC content curves in Fig. 3b were used to compute a temperature score for each site that is higher if sediments were subjected to higher temperatures during burial (see Methods). This alleviates the effect of the choice of activation energy in the Arrhenius rate law; a different value would result in different predicted POC curves, but the ordering of the temperature scores would remain the same. On the basis of the computed temperature score, we selected the 40% of sites that experienced the lowest and highest temperatures during burial, disregarding the middle 20% of sites with intermediate temperature histories. This choice balances the competing needs of obtaining two sets of sites that have distinct predicted POC contents while having as many sites as possible in each set.

Whereas the sites in the low- and high-temperature sets (Fig. 3a) have similar distances from land regions, seafloor depths, and POC content at the seafloor, the sedimentation rates are different. The overall sedimentation rate distributions in each set are approximately lognormal, with a mean log-sedimentation rate of 45 m/Ma in the low-temperature sites compared to 314 m/Ma in the high-temperature sites (Supplementary Figs. 1 and 2). This arises from the effect of sedimentation rate on the temperature history during burial: sites with higher sedimentation rates are more likely to be included in the high-temperature set. Sedimentation rate, however, is one of the factors that can affect POC deposition and preservation, and different POC versus age relationships in the low- and high-temperature sets may be related to differences in sedimentation rate. To account for these differences, we calculate POC averages in the low- and high-temperature sets of sites by applying weights that compensate for the effect of differing sedimentation rates (see Methods).

**Figure 4:**
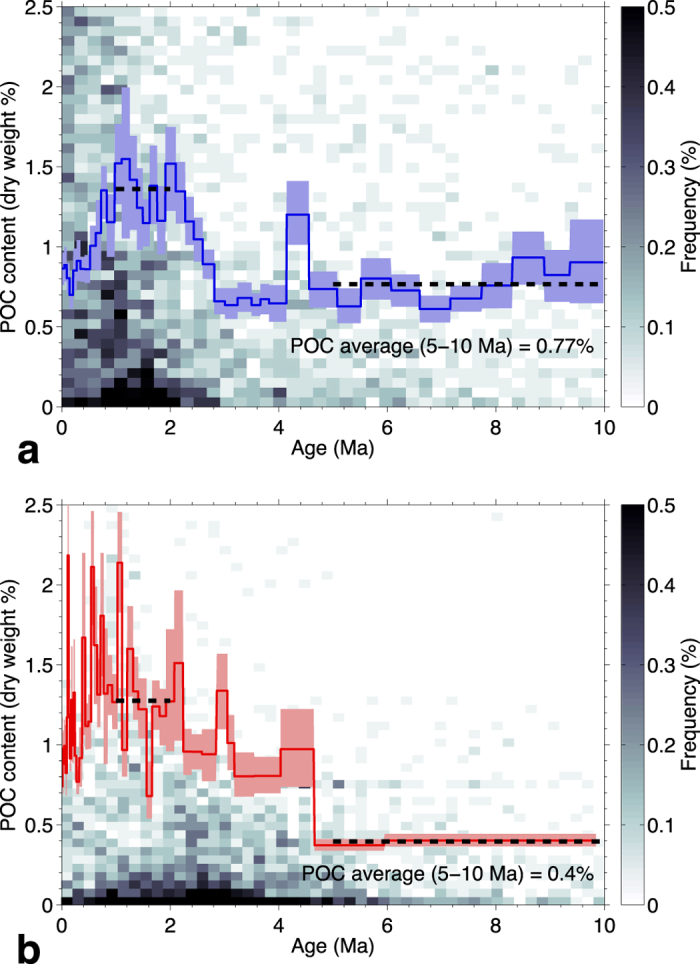
POC in low- and high-temperature sites. POC content versus sediment age in 82 low-temperature sites (**a**) and 83 high-temperature sites (**b**). Background images are histograms of 3104 (**a**) and 3573 (**b**) POC data points, and continuous curves are the average POC in age intervals that contain ~70 data points. The POC averages were computed with weights that compensate for differences in the distribution of sedimentation rates (see Methods). The colored bands around the average are a measure of uncertainty (68% central interval in bootstrap resampling; see Methods). The dashed lines are average POC contents in the 1–2 Ma and 5–10 Ma age intervals. In the two sets of sites, these averages are similar in the 1–2 Ma interval (~1.3%) and are different in the 5–10 Ma interval.

The average measured POC contents versus age in the low- and high-temperature sets of sites are in Fig. 4. The overall pattern in both sets is similar to that observed for all the sites (Fig. 1b); the average POC first increases with age, reaching about 1.3% in the interval 1–2 Ma, then decreases becoming nearly constant for ages 5–10 Ma. This long-term 5–10 Ma POC average is quite different in the two sets, being 0.4% in the high-temperature sites and 0.77%, almost twice as much, in the low-temperature sites.

## Discussion

The more pronounced POC decrease at 5–10 Ma in the high-temperature sites supports the hypothesis that higher temperatures result in a greater extent of POC degradation. To test this conclusion further, we check whether these POC differences are statistically significant or could be due to shortcomings in our data set.

We assess the significance of the differences in average POC at 5–10 Ma with a simple Monte Carlo randomization test. In each of 1000 iterations, two sets of sites are selected at random from the 207 that had reliable geothermal gradients, each set containing 40% of the total number of sites. The average POC content with age is then computed as done for the low- and high-temperature sets of sites, and the difference in the 5–10 Ma average between the two sets is calculated. At the end of the experiment, we compute the fraction of cases where the difference in the 5–10 Ma POC average between the two randomly selected sets of sites is as large as or larger than that observed. This fraction estimates the probability that the observed difference between the low- and high-temperature sets of sites was due to chance. We obtain a relatively small probability of 5.5%, implying that it is unlikely that the difference in the 5–10 Ma POC averages is simply due to the fluctuations of measured POC values in our data set.

This significance level is sensitive to the fraction of sites in the low- and high-temperature sets. If we increase this fraction by including in the two sets the top and bottom 45% of the temperature scores, the difference in the 5–10 Ma POC averages decreases: the averages are 0.77% and 0.61% in the low- and high-temperature sites, respectively. The randomization test accordingly gives a greater probability of 26% for this difference to be due to chance. However, this could be due to having more sites in each set that experienced similar temperature histories, thus reducing the differences in the overall effect of temperature on each set. At the other end, if the two sets of sites are constructed with the top and bottom 35% of the temperature scores, the difference in the 5–10 Ma POC averages remains relatively large (0.59% and 0.28% in the low- and high-temperature sites, respectively). On the other hand, the decrease in the number of sites and of data points in each set increases the variability of the POC averages and the probability that the observed differences in the 5–10 Ma averages are due to chance (13%).

Another effect of the higher sedimentation rates in the high-temperature sites is that this set of sites has relatively few POC data points (~140) at ages >4.5 Ma. It is then possible that the low 5–10 Ma POC average may be due to having only data from sites where POC was low throughout the sediment column. In the nine high-temperature sites that had POC data at ages >4.5 Ma, however, there is no indication that POC is lower than in the whole high-temperature set (Supplementary Fig. 3).

In conclusion, global observations show a more pronounced long-term (5–10 Ma) decrease in POC content where sediments experienced higher temperatures during burial. This difference seems unlikely to be due to differences in sedimentation rate, to the intrinsic variability of the POC data, or to sparser sampling of older sediments. Our results suggest that temperature should be taken into account when predicting and modeling rates of biogeochemical processes in the deep sedimentary biosphere. By enhancing the reactivity of POC, increasing temperatures may result in the degradation of a greater fraction of POC at depth and provide an important source of energy for an active deep biosphere.

## Methods

### Ocean drilling data

The sedimentary POC content, *in situ* temperature measurements, and age data used here were collected between 1968 and 2012 in successive scientific ocean drilling programs (the Deep Sea Drilling Project, the Ocean Drilling Program, and the Integrated Ocean Drilling Program) and are available in online digital databases (International Ocean Discovery Program, http://www.iodp.org/access-data-and-samples, date of access: 20/10/2015) and reports (International Ocean Discovery Program, http://www.iodp.org/scientific-publications, date of access: 20/10/2015). All the measurements of POC and temperature, age data, and age models compiled in this study and described below have been published in an open access data repository^47^.

The carbon content in core samples (dry weight fraction) was estimated by measuring two of three quantities: total carbon (TC), inorganic carbon (IC), and particulate organic carbon (POC), where TC = IC + POC. The POC content was determined in one of two ways: (1) Measuring TC from the CO_2_ generated by sample combustion at very high temperatures and IC (as carbonate carbon) from the CO_2_ liberated by reaction with acid^48^; POC is then given by the difference TC – IC. (2) Directly measuring POC from the total carbon content of a sample previously treated with acid to eliminate carbonate carbon^49^. The latter method is likely to result in more accurate measurements of POC, especially if there is a sizable fraction of carbonate carbon and POC is calculated in the first method from the difference of two large carbon amounts. Based on measurement reproducibility and comparisons of the two techniques^50^,^51^, we estimate the POC measurement uncertainties to be ±0.1–0.25%, with the higher uncertainties for POC computed from the difference TC – IC.

Measurements of *in situ* sediment temperature taken in scientific ocean drill holes are described in ref. 52and data analyses suggest uncertainties in the measured temperatures of 0.1–0.5 °C. The temperature measurements in our data set of 207 sites are summarized in Fig. 2. There are three or more temperature measurements at most sites, and we only included sites where the measurements aligned on a well-defined linear geothermal gradient. A linear geothermal gradient is expected for lithospheric heat flow because variations with depth in the thermal conductivity of sediments are generally minor. Linear geothermal gradients were estimated at each site with a least squares fit. As a further check, we also ensured that the estimated seafloor temperature was consistent with near-bottom ocean temperatures listed in the 2013 NOAA World Ocean Atlas^53^.

The age data were determined from micro- and nannofossil biostratigraphy (first/last appearances of age-diagnostic taxa or biostratigraphic zonation) and from magnetostratigraphy. When possible, age assignments were updated with recent surveys of nannofossil^54^, foraminifera^55^, and magnetic^56^ stratigraphy. Uncertainties of these age assignments are on the order of ~10 ka in the Pleistocene and increase to ~100 ka around 10 Ma. On the basis of these age data, we then constructed age models consisting of depth-age nodes that give the sediment age at any desired depth by linear interpolation^47^.

### Bootstrap uncertainty of POC averages

The POC average curves in Figs 1b and 4a,b are computed in age intervals that contain a nearly constant number of POC data points. We estimated an uncertainty for the computed POC averages with a bootstrap resampling method, which does not require any assumption on the underlying distribution of data values^43^. In each age interval, we computed 1000 averages by resampling with replacement the POC data. The uncertainty bands in Figs 1b and 4a,b was determined as the interval containing the central 68% of the POC averages obtained by resampling. This uncertainty corresponds to the ±1*σ* interval for a normal distribution. The resampled POC averages seem to be nearly normally distributed, because the bootstrap intervals are roughly symmetric about the average and because the 95% bootstrap intervals (±2*σ* for a normal distribution) are approximately twice the 68% intervals.

### Calculation of temperature history and predicted POC content

At each site that had an age model and a reliable estimated geothermal gradient, we first determined the present-day sediment temperatures at 100 ages evenly spaced between 0 and 10 Ma. For each of these age points, we computed from the age model the temperature history experienced during burial between the time the sediment was deposited at the seafloor and the present. This calculation assumed that a linear temperature gradient could be extrapolated to the depth where the sediment age is 10 Ma and that the presently measured temperature gradient remained the same over the last 10 Ma.

The reaction rate constant for POC degradation during burial was defined from the Arrhenius equation as^24^,^25^


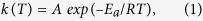


where *A* is a constant, *E*_*a*_ the activation energy of POC degradation, *R* the universal gas constant, and *T* absolute temperature. The activation energy was set to 110 kJ/mole (ref. 57), and the constant *A* was adjusted to result in a range of predicted POC contents for the temperature histories in our data set between 0 and 10 Ma. The final value we used was *A* = 3·10^18^ Ma^–1^.

The fundamental equation describing the change in labile POC content *G* with time *t* for a reaction rate constant *k*(*t*) is^16^,^17^


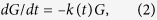


whose solution is, for an initial POC content *G*_*0*_ at *t* = 0,


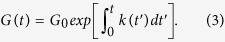


At each of the 100 age points in a site, the temperature history was used to compute a corresponding variation with time of the reaction rate constant *k*(*t*), which was then integrated numerically to compute *G*(*t*) for an initial *G*_0_ = 1. The resulting curves *G*(*t*) of predicted POC content at each site are in Fig. 3b.

To select sets of low- and high-temperature sites, we computed a temperature score *S* at each site from the predicted POC content *G*(*t*) as follows:


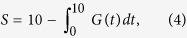


where time *t* is in Ma. If sediment at a site experienced low temperatures during burial, the modeled *G*(*t*) will not decrease much from its initial value of 1 at zero age, the integral of *G*(*t*) in Eq. 4 will be nearly 10, and the score *S* for that site will be low. At the other extreme, the score *S* will be high at a site where sediments underwent high temperatures and where *G*(*t*) decreases rapidly toward zero. Sites with the lowest 40% temperature scores were assigned to the low-temperature set, and sites with the highest 40% scores to the high-temperature set (Fig. 3b). The sites in the low- and high-temperature sets are listed in Supplementary Tables 1 and 2.

### Weighted POC averages that compensate for different distributions of sedimentation rates

We applied a weighting scheme to account for the effect of different distributions of sedimentation rates in the low- and high-temperature sets of sites. The weights are based on the local sedimentation rate at each POC data point and are chosen to effectively make the distribution of sedimentation rate in each set of sites match a target lognormal distribution. The target distribution closely approximates the distribution of sedimentation rates in the 419 sites that have an age model.

Supplementary Figs. 1 and 2 show the sample distribution of sedimentation rates in each set of sites estimated by kernel smoothing^58^, the target distribution, and the computed weights. The weights are set to the ratio between the value of the target and of the sample distribution at the sedimentation rate of each POC data point. Consequently, POC data points with sedimentation rates that are overrepresented in the set are weighed less whereas POC data points with underrepresented sedimentation rates are weighed more. In the low-temperature set of sites (Supplementary Fig. 1), the weights are <1 for sedimentation rates below about 100 m/Ma and are <1 for higher sedimentation rates, giving more importance to POC data points with high sedimentation rate to balance the relatively small fraction of high sedimentation rates in this set. In the high-temperature set (Supplementary Fig. 2), the weights follow the opposite pattern, being >1 for sedimentation rates below about 100 m/Ma and <1 for higher sedimentation rates. To ensure that the calculations are done for the same range of sedimentation rates, we also set to zero the weights for sedimentation rates that were outside the interval spanned by both sets of sites (8.3–2037 m/Ma). The bulk of the data in each set of sites (88.6% to 94.7%) had sedimentation rates within this range.

The average POC contents calculated by applying these weights approximate the averages that would be obtained if the distribution of sedimentation rates in each set of sites was the same as the target distribution and remove the effect of having different sedimentation rate distributions in the low- and high-temperature sets. The average POC contents in Fig. 3a,b were computed by applying these weights.

## Additional Information

**How to cite this article**: Malinverno, A. and Martinez, E. A. The effect of temperature on organic carbon degradation in marine sediments. *Sci. Rep.* **5**, 17861; doi: 10.1038/srep17861 (2015).

## Supplementary Material

Supplementary Information

Supplementary Data set 1

Supplementary Data set 2
