# Supplementary material for: The effect of temperature on organic carbon degradation in marine sediments
Source: Sci Rep. 2015 Dec 7;5:17861. doi: 10.1038/srep17861 (PMC4671099; doi:10.1038/srep17861)
Supplement: Supplementary Information [file srep17861-s1.pdf]

Supplementary Information

The effect of temperature on organic carbon decomposition in marine sediments

Alberto Malinverno[1]* and Ernesto A. Martinez[2†]

[1]Lamont-Doherty Earth Observatory of Columbia University, Palisades, NY, USA.
[2]Department of Earth and Planetary Sciences at the University of California, Berkeley, CA, USA.
*e-mail: alberto@ldeo.columbia.edu.
[†]Current address: 1761 Park Ave., Unit G, Long Beach, CA 90815, USA.

Supplementary Tables 1 and 2. These supplementary tables list the drill holes in the low-temperature sites (Supplementary Table 1) and high-temperature sites (Supplementary Table 2). The columns in the table are as follows:

- holeid: Drilling leg or expedition number followed by hole number;
- lat(deg): Latitude in decimal degrees;
- lon(deg): Longitude in decimal degrees;
- zsf(m): Seafloor depth in meters;
- dist2cont(km): Distance to nearest land area (> 2000 km^2) in km;
- POCsf(%): POC content at the seafloor interpolated from ref. 32 in dry weight %.

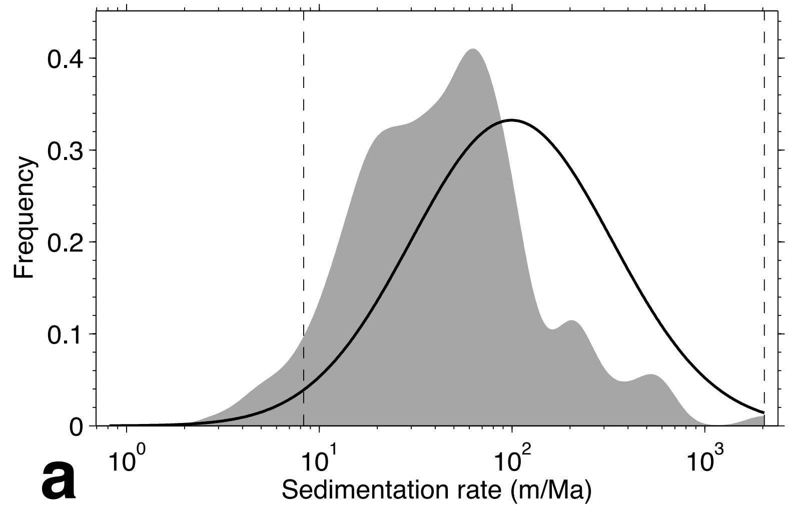

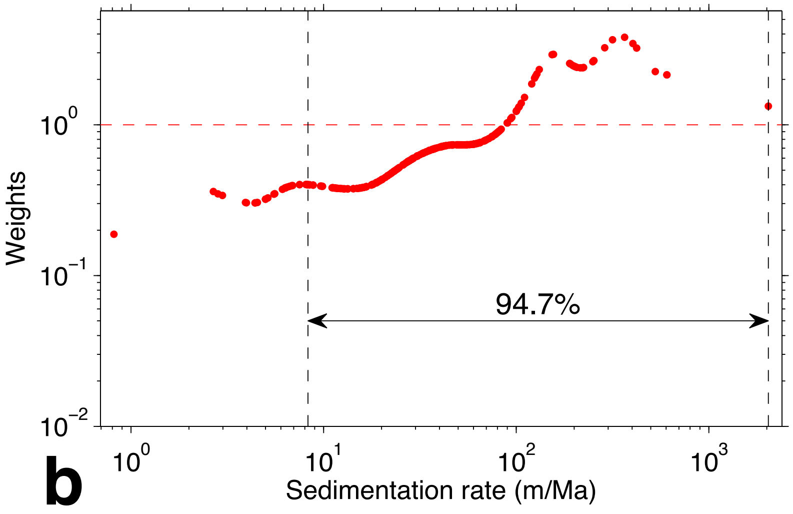

Supplementary Figure 1. Sedimentation rates and weights in the 82 low-temperature sites. Probability distribution of sedimentation rates (**a**) and calculated weights (**b**). The filled gray region in (**a**) is the sample probability distribution estimated by kernel smoothing and the continuous curve is the target distribution, which is a lognormal that approximates closely the distribution of sedimentation rates in the 419 sites that have an age model. The vertical dashed lines in (**a**) and (**b**) denote the range of sedimentation rates spanned by data in both the low- and high-temperature sets of sites (8.3-2037 m/Ma); 94.7% of all sedimentation rates in the low-temperature set are within this range.

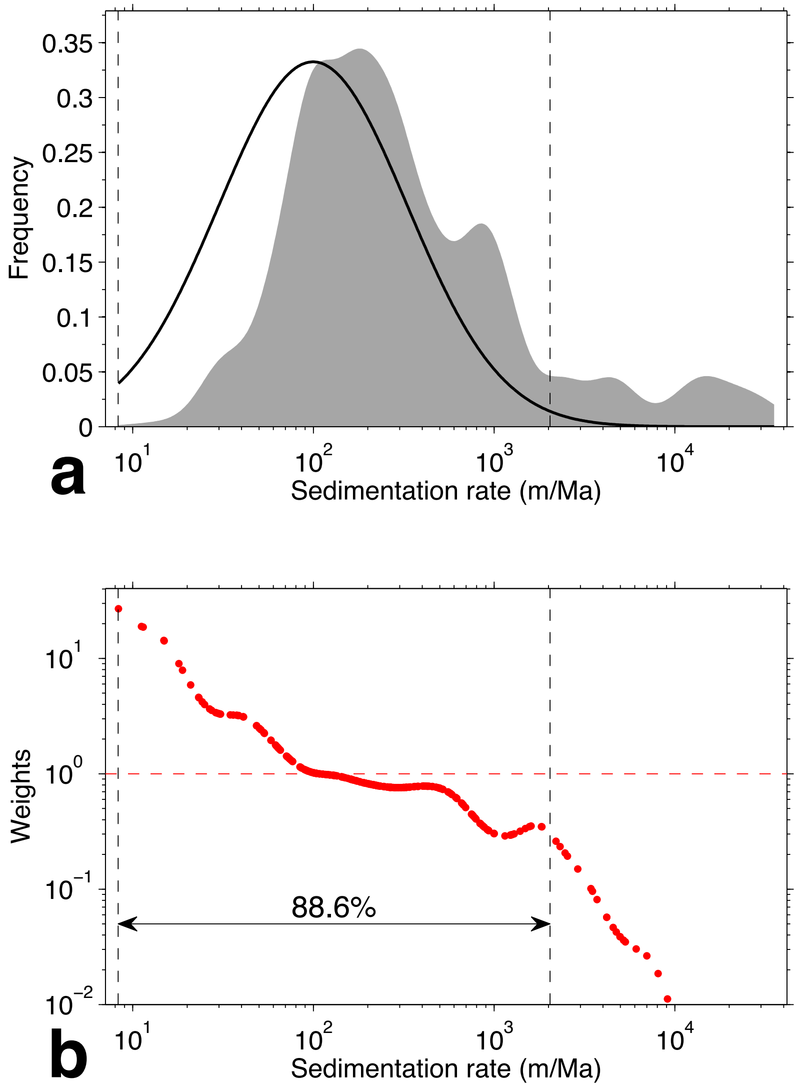

Supplementary Figure 2. Sedimentation rates and weights in the 83 high-temperature sites. Symbols as in Supplementary Fig. 1. 88.6% of all sedimentation rates in the high-temperature set are within the range of sedimentation rates spanned by data in both sets of sites (8.3-2037 m/Ma).

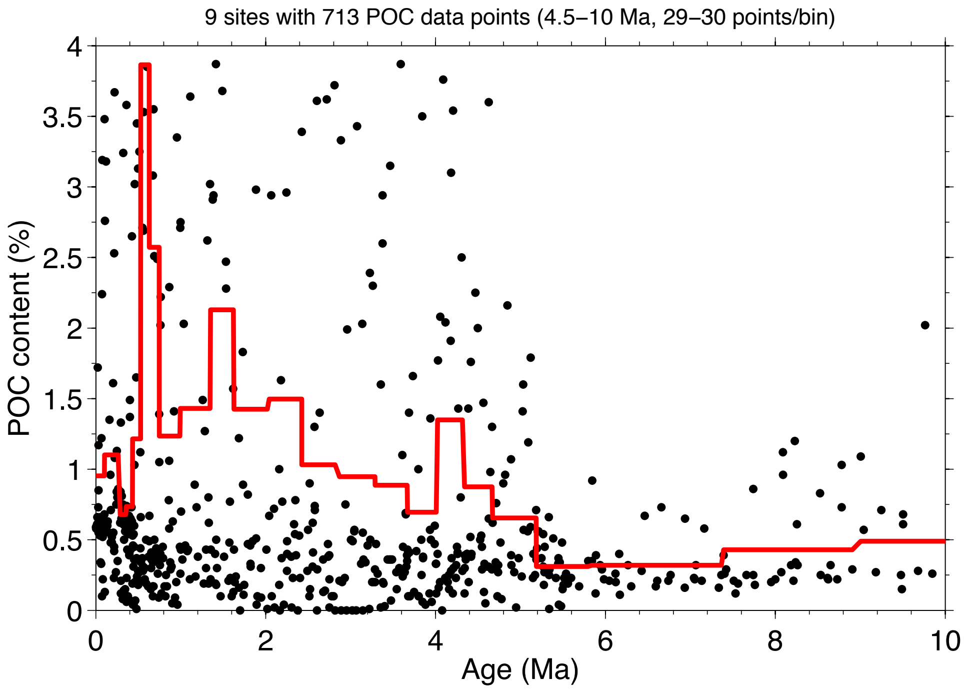

Supplementary Figure 3. POC content versus age in the nine high-temperature sites that have data for ages > 4.5 Ma. The nine sites plotted are Sites 225 and 227 (DSDP Leg 23, Red Sea), Site 677 (ODP Leg 111, Costa Rica rift), Site 795 (ODP Leg 127, Japan Sea), Site 808 (ODP Leg 131, Nankai Trough), Site 833 (ODP Leg 134, Vanuatu, New Hebrides), Site 1082 (ODP Leg 175, Benguela Current, Namibia), Sites 1173 and 1174 (ODP Leg 190, Nankai Trough). POC measurements are shown as black dots, and the continuous line is the average calculated in age intervals that contain the same number of data points (~30). POC measurements > 4% are not plotted to facilitate comparison with Fig. 4b.
